# Screening of *Klebsiella pneumoniae* subsp. *pneumoniae* Strains with Multi-Drug Resistance and Virulence Profiles Isolated from an Italian Hospital between 2020 and 2023

**DOI:** 10.3390/antibiotics13060561

**Published:** 2024-06-15

**Authors:** Valentina Dimartino, Carolina Venditti, Francesco Messina, Silvia D’Arezzo, Marina Selleri, Ornella Butera, Carla Nisii, Alessandra Marani, Alessia Arcangeli, Roberta Gaziano, Terenzio Cosio, Pietro Scanzano, Carla Fontana

**Affiliations:** 1 National Institute for Infectious Diseases Lazzaro Spallanzani IRCCS, 00149 Rome, Italy; valentina.dimartino@inmi.it (V.D.); carolina.venditti@inmi.it (C.V.); francesco.messina@inmi.it (F.M.); silvia.darezzo@inmi.it (S.D.); marina.selleri@inmi.it (M.S.); ornella.butera@inmi.it (O.B.); carla.fontana@inmi.it (C.F.); 2 Health Direction, National Institute for Infectious Diseases Lazzaro Spallanzani IRCCS, 00149 Rome, Italy; alessandra.marani@inmi.it (A.M.); alessia.arcangeli@inmi.it (A.A.); pietro.scanzano@inmi.it (P.S.); 3 Department of Experimental Medicine, University of Rome Tor Vergata, Via Montpellier 1, 00133 Rome, Italy; roberta.gaziano@uniroma2.it; 4 Dermatology Unit, Department of Systems Medicine, Tor Vergata University Hospital, 00133 Rome, Italy; terenziocosio@gmail.com

**Keywords:** hypervirulent strains, multi-drug resistant, hospital-acquired infections, convergence of virulence and antimicrobial resistance

## Abstract

*Klebsiella pneumoniae* strains that are resistant to multiple drugs (KPMDRs), which are often acquired in hospital settings and lead to healthcare-associated infections, pose a serious public health threat, as does hypervirulent *K. pneumoniae* (hvKp), which can also cause serious infections in otherwise healthy individuals. The widespread and often unnecessary use of antibiotics seen during the recent COVID-19 pandemic has exacerbated the challenges posed by antibiotic resistance in clinical settings. There is growing concern that hypervirulent (hvKp) strains may acquire genes that confer antimicrobial resistance, thus combining an MDR profile with their increased ability to spread to multiple body sites, causing difficult-to-treat infections. This study aimed to compare resistance and virulence profiles in KPC-3-producing *K. pneumoniae* isolates collected over four years (2020–2023). A genome-based surveillance of all MDR CRE-*K. pneumoniae* was used to identify genetic differences and to characterize the virulence and resistance profiles. Our results provide a picture of the evolution of resistance and virulence genes and contribute to avoiding the possible spread of isolates with characteristics of multi-drug resistance and increased virulence, which are thought to be one of the main global challenges to public health, within our hospital.

## 1. Introduction

Carbapenem-resistant Enterobacteriales (CREs), most notably carbapenem-resistant (CR) *Klebsiella pneumoniae*, are among the main causes of severe hospital-acquired bacterial infections worldwide [1]. In Italy, the predominant mechanism of carbapenem resistance is the production of KPC serine-beta lactamases [2] with an overall prevalence of so-called high-risk clones (e.g., members of clonal groups (CGs) 258, 101, 147, and 307). According to a recent report by the European Centre for Disease Prevention and Control (ECDC), the incidence of CR-*K. pneumoniae* increased by almost 50% between 2019 and 2022 in Europe, while in Italy, their spread seems to be even higher: 92.8% of all CR-K. pneumoniae were identified as KPC-K.pneumoniae producers [3,4]. In this scenario, the additional strain posed by the COVID-19 emergency on the national health system was likely to increase the risk of developing superinfections by multi-drug-resistant (MDR) pathogens, as confirmed by several reports of hospital outbreaks [5,6,7]. One of the main causes of this trend could be an overuse of broad-spectrum antibiotics to fight severe bacterial infections in settings such as surgical wards and intensive care units [8], as also reported by the WHO through their Global Clinical Platform for COVID-19 [9,10]. Furthermore, the ECDC issued an alert in 2021 [11] to raise awareness of the possible spread of hypervirulent KP (hvKp) strains. While hvKp bacteria have been widely described as susceptible to antibiotics, they show a higher rate of occurrence in the community and a capability of infecting healthy individuals [12]. Although hvKp is rarely MDR, the ECDC warned about the possibility that strains of hvKp could become a serious health threat if combined with carbapenem resistance. This threat represents a serious concern for hospital settings, and various surveillance studies have been published, aimed at assessing the prevalence of hvKp in general and, more specifically, of MDR-hvKp [13,14,15].

Following the ECDC alert, and concerned by the additional burden of dealing with the COVID-19 emergency, we implemented a hospital-wide genomic surveillance of CR-*K. pneumoniae* strains isolated in our hospital that included screening for virulence-associated genes. Also, strains collected in 2020 and the earlier months of 2021 (the peak of the COVID-19 period) were retrospectively analyzed. This study aimed to describe the epidemiological and molecular resistance and virulence features of KPC-*K. pneumoniae* strains collected between 2020 and January 2023 to highlight any changing patterns of virulence and/or antibiotic resistance. Single-event transfer of drug resistance genes and virulence genes have been reported, so monitoring of this phenomenon appears to be necessary [16,17,18].

## 2. Results

### 2.1. Phenotypic Characteristics

Sixty CR-*K. pneumoniae* isolates were collected from 60 critically ill patients treated in the ICU during the study period to investigate potential resistance and virulence evolution profiles. As shown in Table S1 (Supplementary Data), isolates were obtained from different specimens, including 29 rectal swabs, 12 blood cultures, 12 bronchial–alveolar lavages (BALs), and 7 urine samples. We computed the crude prevalence of carbapenem-resistant strains per year from June 2020 to December 2022, using only the first isolate per patient. The observed prevalence rates were 3.8% in 2020 (5 out of 131 isolates), 10.8% in 2021 (14 out of 129 isolates), and 12.25% in 2022 (31 out of 253 isolates).

All strains were resistant to carbapenems except strain 2022-KPC-Kpn-21, which lacked the *bla*_KPC_ gene and exhibited elevated MIC values for carbapenems and was close to the resistance threshold according to the most recent EUCAST criteria [19]. All CR-*K. pneumoniae* strains were KPC-3-variant producers except for one carrying KPC-19, as shown in Table S1 (Supplementary Data). One strain, carrying KPC-3 (2022-KPC-Kpn-39), was susceptible to meropenem but resistant to both imipenem and cefiderocol. Five more isolates showed resistance to cefiderocol (2021-KPC-Kpn-9, 2022-KPC-Kpn-29, 2022-KPC-Kpn-40, 2022-KPC-Kpn-48, and 2022-KPC-Kpn-50). All strains were susceptible to ceftazidime/avibactam except for 2022-KPC-Kpn-29, which carried the KPC-19 allele. Some strains also showed resistance to aminoglycosides, and all isolates were generally quinolone-resistant, except for 2023-KPC-Kpn-58. Two strains were resistant to colistin (2021-KPC-Kpn-9 and 2023-KPC-Kpn-57). The string test, performed according to Hagiya et al, was negative on all tested isolates, and none showed a hypermucoviscous phenotype [20].

### 2.2. Genomic Characterization of Virulence and Resistance Profiles in KP Isolates

All strains were analyzed by whole-genome sequencing (WGS), and the results are summarized in Table 1.

Two strains isolated in 2020 (2020-KPC-Kpn-3 and 2020-KPC-Kpn-5) showed a limited number of resistance genes: *bla*_KPC-3_ and *bla*_SHV-1_. All the other strains (except the already mentioned 2022-KPC-Kpn-20) carried an array of genes conferring widespread resistance to β-lactams and various other antibiotics. Analyzing the data obtained from 2020 to 2023, we noticed changing patterns of resistance determinants; for example, the most prevalent ST512 exhibited *bla*_OXA-9_, *bla*_SHV-11_, and *bla*_TEM-1D_ genes in strains isolated from 2020 to 2021, while in 2022 and 2023, the most frequently observed β-lactam resistance genes were *bla*_KPC-3_, *bla*_CMY-16_, *bla*_OXA-10_, *bla*_SHV-11_, and *bla*_TEM-1D_.

**Figure 1:**
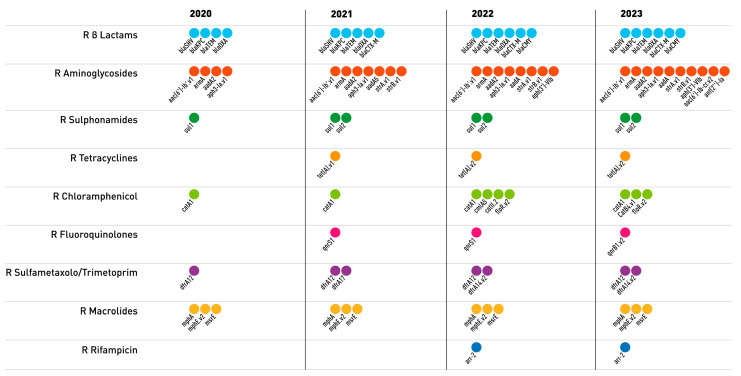
Each dot represents the resistance gene for the specific class of antibiotic, divided by year.

The changing pattern of resistance genes was also evident when analyzing their distribution by class of antibiotics, as shown in Figure 1; especially for β-lactams and aminoglycosides, our results show an increase in the number of genes conferring resistance, especially in 2023.

**Figure 2:**
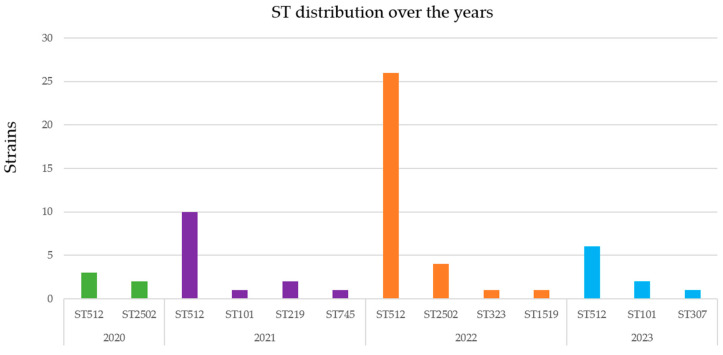
Distribution of STs during the period of observation.

A total of 8 different sequence types (STs) were identified: ST512 (n = 45), ST2502 (n = 6), ST307 (n = 1), ST323 (n = 1), ST219 (n = 2), ST1519 (n = 1), ST745 (n = 1), and ST101 (n = 3). ST512 was found to be the most prevalent (45 isolates—75%), followed by ST2502 (6 isolates—10%), ST101 (3 isolates—5%), and ST219 (2 isolates—3%); ST1519, ST307, ST323, and ST745 were represented by only one isolate each and altogether accounted for 2% of the total. The distribution of STs identified between 2020 and 2023 is shown in Figure 2: ST512 was consistently found to be the most represented, with a peak in 2022, while others (ST745 and ST219) appeared briefly in 2021 and disappeared in 2022 and 2023, giving way to ST307, ST323, and ST519. The years 2020 and 2021 (the peak of the COVID-19 period) were the ones that showed the most diversity of isolated STs.

**Figure 3:**
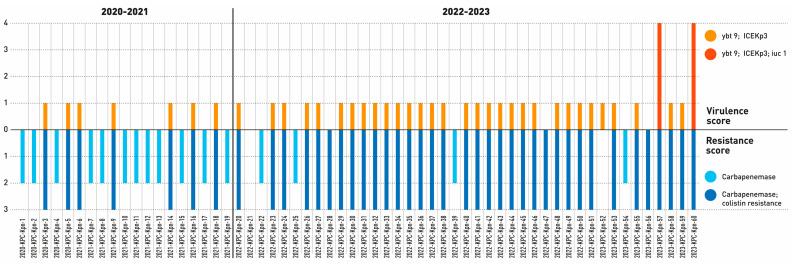
Distribution of virulence and resistance scores over the years under investigation. The orange lines represent the virulence scores and the blue lines values of the resistance scores based on the data generated by Kleborate software.

Our study also addressed the presence of genes associated with virulence, assigning a score from 0 to 5 [21]; analyzing the data obtained from 2020 to 2021, we found that 36% of the strains exhibited a virulence score of 1, owing to the presence of the yersiniabactin gene. From 2022 to 2023, 75% of the strains showed a virulence score of 1, and two isolates (2023-KPC-Kpn-57 and 2023-KPC-Kpn-60) reached a score of 4, carrying yersiniabactin (ybt) and aerobactin (iuc1) genes and (in one case) the rmpA2 gene (Table 1). Both strains belonged to ST101. Likewise, we also observed an increase in the resistance scores between 2020 and 2023 (Figure 3).

**Figure 4:**
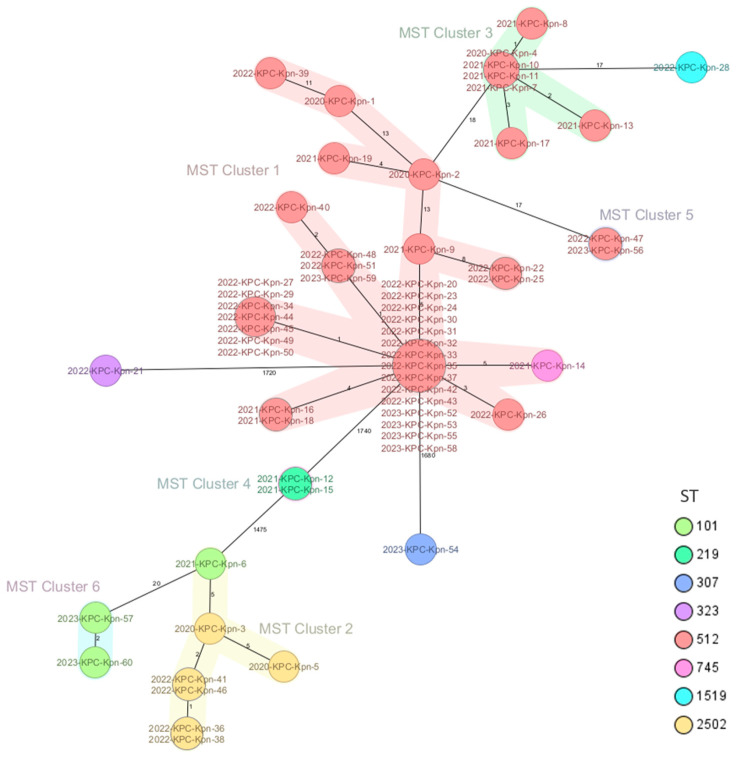
The numbers indicate the allelic distance between different strains or clusters based on cgMLST analysis with RIDOM SEQSPHERE software. MST cluster distance threshold: 15. ST: sequence type.

By using WGS and Ridom SeqSphere+ software version 9.0.10 2023-09 and according to cgMLST results, the isolates were found to belong to six different cluster types (CTs), CT-1 to CT-6 (Figure 4). CT-1 included 37 isolates, all belonging to ST512 except 1 (2021- KPC-Kpn-14) which belonged to ST745. CT-2 comprised six ST2502 isolates and one ST101 (2021- KPC-Kpn-6). Seven ST512 isolates were grouped in CT-3. CT-4 included two ST219 strains (2021-KPC-Kpn-12 and 2021-KPC-Kpn-15). Two isolates (2022-KPC-Kpn-47 and 2023-KPC-Kpn-56), both ST512, formed CT-5. CT-6 included two strains belonging to ST101 (2023-KPC-Kpn-57 and 2023-KPC-Kpn-60). Three isolates were not found to belong to any cluster: 2022-KPC-Kpn-21, 2022-KPC-Kpn-28, and 2023-KPC-Kpn-54, belonging to ST323, ST1519, and ST307, respectively.

## 3. Discussion

Antimicrobial resistance and the emergence of CR-*K. pneumoniae* strains represent a major problem in hospitals worldwide. The recent SARS-CoV-2 pandemic has placed an additional burden on healthcare facilities and their ability to control the spread of antimicrobial resistance [22,23,24]. In ICUs in particular, the risk of MDR infections increased consistently owing to the high number of invasive procedures, such as mechanical ventilation, and the use of central venous catheters [25,26,27,28] and because of the intensive use as well as the overuse of broad-spectrum antibiotics. The warning received from the ECDC in 2021 [11] on the possible emergence of hvKp strains carrying determinants of multi-drug resistance caused further concern about the potential spread of isolates in which the convergence of virulence and antimicrobial resistance may pose an even greater threat to healthcare systems [29,30,31]. This prompted us to strengthen our surveillance activities and include a search for determinants of virulence in addition to those for drug resistance [32,33]. To this end, we carried out WGS on 60 strains of CR-*K. pneumoniae* collected between 2020 and 2023, a period that included the peak months of the COVID-19 pandemic, i.e., the time when our hospital was suffering an exceptional burden of critical patients, having been designated as the main COVID-19 hospital in the Latium region. Our findings showed a combination of genes conferring resistance, mainly to β-lactams and aminoglycosides, throughout the study period (Figure 1), with a slight increase in 2022–2023; resistance to cefiderocol was sporadic and mainly present in 2022–2023; one strain showed resistance to ceftazidime/avibactam as well as cefiderocol (2022-KPC-Kpn-29) (Table 1). The predominant resistance mechanism can be ascribed to the presence of KPC-type 3 in all isolates except for one strain that carried KPC-19 (2022-KPC-Kpn-29).

A wide range of STs were identified, some more frequently than others, in particular, ST512 (Figure 2). The predominance of ST512 was a constant feature throughout the study period but was particularly evident in 2022, owing to a higher number of cases of in-hospital transmission that were readily identified and controlled. ST512 was also found to be the most represented in a previous study conducted during a one-year survey of ceftazidime–avibactam-resistant *K. pneumoniae* collected from six different hospitals in Rome [34], and it was also shown to be one of the most widely circulating and highly conserved clones in the country [35].

Given the importance of monitoring the circulation of hvKp that are also MDR [13,14,15,36], our study also focused on determinants of virulence since the spread of hvKp strains has been observed in specific settings and/or in patients with severe SARS-CoV-2 infections [37,38,39]. Overall, we observed a higher number of strains carrying virulence determinants in 2022–2023, although with low scores (Figure 3); only two strains had a virulence score of 4 (due to the acquisition of *iuc1* and *ybt*), both isolated in 2023, and both belonging to ST101 (Table 1 and Figure 3). This higher virulence profile observed in ST101 is noteworthy since ST101 has been recognized as an emerging clone in different parts of the world, carrying an 11% increase in mortality rates compared to infections with non-ST101 clones [40].

Moreover, in 2023, one isolate (2023-KPC-Kpn-60) also carried the *rmpA2* gene, which is involved in a hypermucosal phenotype, although the string test was negative for this isolate [41]; this is not surprising, as the performance of the string test has also been shown to be inconsistent in other studies [14,15], highlighting the importance of a combined phenotypic, molecular, and WGS approach even more. Ridom SeqSphere+ software version 9.0.10 2023-09 was used to investigate the presence of clusters of transmission. We identified six clusters (CTs) and three unrelated strains (Figure 4). CT-1 was the largest and included strains collected throughout the study period, many belonging to ST512. This cluster included a strain belonging to ST745. The latter has been reported as a single locus variant (SLV) of ST512 [42]. The two strains with increased virulence scores that were collected in 2023 were both part of CT-6. The remaining CTs comprised strains collected during the whole study period (Figure 3).

## 4. Materials and Methods

The ‘L. Spallanzani’ National Institute for Infectious Diseases in Rome is a care and research facility dedicated to infectious diseases that was designated as the main COVID-19 hospital of the Latium Region. During a time frame that included the peak of the COVID-19 emergency, (from June 2020 to January 2023), we performed >2000 cultures and collected 60 CR-*K. pneumoniae* strains. The strains were obtained from different specimens, including 29 rectal swabs, 12 blood cultures, 12 bronchial–alveolar lavages (BALs), and 7 urine samples. Species identification and antimicrobial susceptibility were obtained by MALDI-TOF MS (Version 4.1.100.10, Bruker Daltonics, Bremen, Germany) and a semi-automated Phoenix system (Becton Dickinson Diagnostics, San Diego, CA, USA). As per EUCAST recommendations, MICs for colistin, ceftazidime/avibactam, and cefiderocol were confirmed by the broth microdilution method using the ComASP^®^ system, while for meropenem/vaborbactam, the MTS™ MIC Test Strip (Liofilchem, Roseto degli Abruzzi, Italy) was used. Results were interpreted according to the European Committee on Antimicrobial Susceptibility Testing (EUCAST) guidelines. First, detection and identification of KPC, VIM, IMP, NDM, and OXA-48-like carbapenemases were achieved using immunochromatographic assay (NG-Test CARBA 5, Biotech, Guipry, France) and confirmed by sequencing of the whole genome (WGS) through an Illumina MiSeq instrument (Illumina Inc., San Diego, CA, USA). All raw reads generated were submitted to the Sequence Read Archive (SRA) under the BioProject ID PRJNA1078116. Resistance and virulence profiles, as well as the determination of sequence types, were obtained using Kleborate software v2.2.0 (https://github.com/katholt/kleborate, accessed on 11 September 2023). Kleborate can identify five specific virulence loci that are often associated with invasive infections and are commonly found in hvKp strains: the siderophores yersiniabactin (*ybt*), aerobactin (*iuc*), and salmochelin (*iro*); the genotoxin colibactin (*clb*); and the hypermucoidy locus *rmp*ADC, the latter of which is associated with a hypermucosal phenotype [43]. Kleborate also provides a simple categorical virulence score (ranging from 0 to 5) that facilitates the monitoring of the convergence of resistance and virulence [21,44]. Genetic relationships were further investigated by the validated WGS-based core genome MLST (cgMLST) using Ridom SeqSphere+ software version 9.0.10 2023-09 (Ridom GmbH, Münster, Germany) with default settings. A gene-by-gene approach comprising 2358 target genes was used to compare genomes based on the defined *K. pneumoniae* sensu lato cgMLST [45] compared to the reference strain (GenBank accession no. NC_012731). The resulting set of target genes was used for interpreting the clonal relationships displayed in a minimum spanning tree; genotypically related isolates (with a cluster distance threshold of 15 alleles) were identified within a Complex Type (CT) (https://www.cgmlst.org, accessed on 15 January 2024). 

## 5. Conclusions

In conclusion, our study remarks on the importance of WGS as a tool to identify the potential determinants of virulence and drug resistance of emerging pathogens and/or convergent phenotypes. In our setting, which was one that saw a rise in antibiotic use due to our role as the main COVID-19 hospital in the region, two ‘convergent’ strains (i.e., strains that were multi-drug-resistant and hypervirulent, therefore being of particular concern) were only identified in 2023, and the fact that no outbreak was subsequently observed suggests that our surveillance was successful in controlling their spread. Limitations of our study are the relatively low number of isolates (collected in a single hospital) and the lack of correlations with the clinical outcomes of patients, which would provide insight into the clinical risk connected with these high-threat isolates. Broader studies involving more than one clinical center and with extensive WGS-based surveillance could shed some light on the real impact of multi-drug-resistant isolates that are also hypervirulent, which may potentially become the next global challenge to public health.

## Figures and Tables

**Table 1:** Molecular characterization of *K. pneumoniae* clinical isolates during the study period (2020–2023).

Resistance Determinants				Virulence Setup		Typing	
Strain	β-Lactam	Additional Resistance Genes	Resistance Score	Virulence Genes	Virulence Score	ST ^1^	CT ^2^
2020-KPC-Kpn-1	*bla*_KPC-3_, *bla*_OXA-9,_*bla*_SHV-11,_*bla*_TEM-1D_	*aac (6’) -Ib fosA_3 oqxA, oqxB*	2	-	0	ST512	1
2020-KPC-Kpn-2	*bla*_KPC-3_, *bla*_OXA-9,_ *bla*_SHV-11,_ *bla*_TEM-1D_	*aac (6’) -Ib, aadA2, aph (3’) -Ia, sul1catA1, fosA_3, qacE, oqxA, oqxB, dfrA12, mph(A)*	2	-	0	ST512	1
2020-KPC-Kpn-3	*bla*_KPC-3_, *bla*_SHV-11_	*msr(E), mph(E), armA, fosA_3, fosA_6, oqxA, oqxB*	3	ybt 9; ICEKp3	1	ST2502	2
2020-KPC-Kpn-4	*bla*_KPC-3_, *bla*_OXA-9,_*bla*_SHV-11,_ *bla*_TEM-1D_	*aac (6’) -Ib, aadA2, aph (3’) -Ia, sul1, catA1, fosA_3, qacE, oqxA, oqxB, dfrA12, mph(A)*	2	-	0	ST512	3
2020-KPC-Kpn-5	*bla*_KPC-3_, *bla*_SHV-1_	*fosA_3, fosA, oqxA, oqxB, armA, _6, msr(E), mph(E)*	3	ybt 9; ICEKp3	1	ST2502	2
2021-KPC-Kpn-6	*bla*_KPC-3_, *bla*_SHV-1,_*bla*_TEM-1D_	*aph(6)-Id, aph(3’)-Ib, aadA5, armA, sul1, sul2, tet(A), fosA_3, qacE, oqxA, oqxB, dfrA14, msr(E), mph(A), mph(E)*	3	ybt 9; ICEKp3	1	ST101	2
2021-KPC-Kpn-7	*bla*_KPC-3_, *bla*_OXA-9,_*bla*_SHV-11,_ *bla*_TEM-1D_	*aac (6’) -Ib, aadA2, aph (3’) -Ia, sul1, catA1, fosA_3, qacE, oqxA, oqxB, dfrA12, mph(A)*	2	-	0	ST512	3
2021-KPC-Kpn-8	*bla*_KPC-3_, *bla*_OXA-9,_ *bla*_SHV-11,_ *bla*_TEM-1D_	*fosA_3, aac (6’) -Ib, aadA2, aph (3’) -Ia, sul1, catA1, qacE, oqxA, oqxB, dfrA12, mph(A)*	2	-	0	ST512	3
2021-KPC-Kpn-9	*bla*_KPC-3_, *bla*_OXA-9,_ *bla*_SHV-11,_ *bla*_TEM-1D_	*sul1, mph(A), aadA2, aph (3’) -Ia, catA1, fosA_3, qacE, oqxA, oqxB, dfrA12*	3	ybt 9; ICEKp3	1	ST512	1
2021-KPC-Kpn-10	*bla*_KPC-3_, *bla*_OXA-9,_ *bla*_SHV-11,_ *bla*_TEM-1D_	*mph(A), aac (6’) -Ib, aadA2, aph(3’)-Ia, sul1, catA1, fosA_3, qacE, oqxA, oqxB, dfrA12*	2	-	0	ST512	3
2021-KPC-Kpn-11	*bla*_KPC-3_, *bla*_OXA-9_, *bla*_SHV-11,_ *bla*_TEM-1D_	*aac (6’) -Ib, aadA2, aph (3’) -Ia, sul I, catA1, fosA_3, qacE, oqxA, oqxB, dfrA12, mph(A)*	2	-	0	ST512	3
2021-KPC-Kpn-12	*bla*_KPC-3_, *bla*_CTX-M-15,_*bla*_SHV-1,_ *bla*_TEM-1D_	*fosA_3, qacE, oqxA, oqxB, qnrS1, dfrA12, mph(A), aph (6) -Id, aph (3’) -Ib, aadA2, sul I, sul2*	2	-	0	ST219	4
2021-KPC-Kpn-13	*bla*_KPC-3_, *bla*_OXA-9,_*bla*_SHV-11,_ *bla*_TEM-1D_	*mph(A), aac (6’) -Ib, aadA2, aph (3’) -Ia, sul1, catA1, fosA_3, qacE, oqxA, oqxB, dfrA12*	2	-	0	ST512	3
2021-KPC-Kpn-14	*bla*_KPC-3_, *bla*_OXA-9,_ *bla*_SHV-11,_ *bla*_TEM-1D_	*fosA_3, oqxA, oqxB*	3	ybt 9; ICEKp3	1	ST745	1
2021-KPC-Kpn-15	*bla*_KPC-3_, *bla*_CTX-M-15,_ *bla*_SHV-1,_ *bla*_TEM-1D_	*fosA_3, qacE, oqxA, oqxB, qnrS1, dfrA12, mph(A), aph (6) -Id, aph(3’)-Ib, aadA2, sul1, sul2*	2	-	0	ST219	4
2021-KPC-Kpn-16	*bla*_KPC-3_, *bla*_OXA-9,_ *bla*_SHV-11,_ *bla*_TEM-1D_	*catA1, fosA_3, qacE, oqxA, oqxB, dfrA12, mph(A), aadA2, aph (3’) -Ia, sul1*	3	ybt 9; ICEKp3	1	ST512	1
2021-KPC-Kpn-17	*bla*_KPC-3_, *bla*_OXA-9,_*bla*_SHV-11,_ *bla*_TEM-1D_	*aac(6’)-Ib, aadA2, aph(3’)-Ia, sul1, catA1, fosA_3, qacE, oqxA, oqxB, dfrA12, mph(A)*	2	-	0	ST512	3
2021-KPC-Kpn-18	*bla*_KPC-3_, *bla*_OXA-9,_*bla*_SHV-11,_ *bla*_TEM-1D_	*catA1, fosA_3, qacE, oqxA, oqxB, dfrA12, mph(A), aac (6’) -Ib, aadA2, aph (3’) -Ia, sul1*	3	ybt 9; ICEKp3	1	ST512	1
2021-KPC-Kpn-19	*bla*_KPC-3_, *bla*_OXA-9_, *bla*_SHV-11,_ *bla*_TEM-1D_	*catAI, fosA_3, qacE, oqxA, oqxB, dfrA12, mph(A), aac (6’)-Ib, aadA2, aph (3’)-Ia, sul1*	2		0	ST512	1
2022-KPC-Kpn-20	*bla*_KPC-3_, *bla*_CMY-16,_ *bla*_OXA-10,_ *bla*_SHV-11_, *bla*_TEM-1D_	*aadA, aadA2, aph (3’)-VIb, aph3-Ia, sul1, sul2, tet(A), floR, catA1, cmlA5, mphA*	3	ybt 9; ICEKp3	1	ST512	1
2022-KPC-Kpn-21	*bla*_SHV-1_	*aadA, sul1*	0	-	0	ST323	-
2022-KPC-Kpn-22	*bla*_KPC-3,_ *bla*_SHV-11_	*aac (6’)-Ib’, aadA2, aph3-Ia, sul I, catA1, mphA*	2	-	0	ST512	1
2022-KPC-Kpn-23	*bla*_KPC-3,_ *bla*_SHV-11,_ *bla*_TEM-1D_	*mphA, aadA2, aph3-Ia, sul1, catA1*	3	ybt 9; ICEKp3	1	ST512	1
2022-KPC-Kpn-24	*bla*_KPC-3,_ *bla*_SHV-11,_ *bla*_TEM-1D_	*catA1, aadA2, aph3-Ia, sul1, mphA*	3	ybt 9; ICEKp3	1	ST512	1
2022-KPC-Kpn-25	*bla*_KPC-3,_ *bla*_SHV-1_	*aac(6’)-Ib’, aadA2, aph3-Ia, sul1, catA1, mphA*	2	-	0	ST512	1
2022-KPC-Kpn-26	*bla*_KPC-3_, *bla*_CTX-M-15,_ *bla*_SHV-11,_ *bla*_TEM-1D_	*aadA2, aph3-Ia, strA, strB, sul1, sul2, catA1, catII.2, mphA*	3	ybt 9; ICEKp3	1	ST512	1
2022-KPC-Kpn-27	*bla*_KPC-3_, *bla*_SHV-11,_ *bla*_TEM-1D_	*aadA2, aph3-Ia, sul I, catA1, mphA*	3	ybt 9; ICEKp3	1	ST512	1
2022-KPC-Kpn-28	*bla*_KPC-3_, *bla*_SHV-11_	*aac(6’)-Ib’, aadA2, aph3-Ia, sul1, catA1, mphA*	3	-	0	ST1519	-
2022-KPC-Kpn-29	*bla*_KPC-19_, *bla*_SHV-11,_ *bla*_TEM-1D_	*aadA2, aph3-Ia, sul1, catA1, mphA*	3	ybt 9; ICEKp3	1	ST512	1
2022-KPC-Kpn-30	*bla*_KPC-3,_ *bla*_CMY-16,_ *bla*_OXA-10,_ *bla*_SHV-11,_ *bla*_TEM-1D_	*aadA, aadA, aadA2, aph (3’)-VIb, aph3-Ia, strA, strB, sul1, sul2, tet(A), floR, catA1, cmlA5, mphA*	3	ybt 9; ICEKp3	1	ST512	1
2022-KPC-Kpn-31	*bla*_KPC-3_, *bla*_CMY-16,_ *bla*_OXA-10,_ *bla*_SHV-11,_ *bla*_TEM-1D_	*aadA, aadA2, aph (3’)-VIb, aph3-Ia, strA, sul I, sul2, tet(A), floR, catA1, cmlA5*	3	ybt 9; ICEKp3	1	ST512	1
2022-KPC-Kpn-32	*bla*_KPC-3,_ *bla*_CMY-16,_ *bla*_OXA-10,_ *bla*_SHV-11,_ *bla*_TEM-1D_	*aadA, aadA2, aph (3’)-VIb, aph3-Ia, strA, sul1, sul2, tet(A), floR, catA1, cmlA5, mphA*	3	ybt 9; ICEKp3	1	ST512	1
2022-KPC-Kpn-33	*bla*_KPC-3_, *bla*_CMY-16,_ *bla*_OXA-10,_ *bla*_SHV-11,_ *bla*_TEM-1D_	*aadA, aadA2, aph (3’)-VIb, aph3-Ia, strA, strB, sul1, sul2, tet(A), floR, catA1*	3	ybt 9; ICEKp3	1	ST512	1
2022-KPC-Kpn-34	*bla*_KPC-3_, *bla*_SHV-11,_ *bla*_TEM-1D_	*aadA2, aph3-Ia, sul1, catA1, mphA*	3	ybt 9; ICEKp3	1	ST512	1
2022-KPC-Kpn-35	*bla*_KPC-3_, *bla*_CMY-16_, *bla*_OXA-10,_ *bla*_SHV-11,_ *bla*_TEM-1D_	*aadA, aadA2, aph (3’)-VIb, aph3-Ia, strA, sul1, sul2, tet(A), floR, catA1, cmlA5, mphA*	3	ybt 9; ICEKp3	1	ST512	1
2022-KPC-Kpn-36	*bla*_KPC-3_, *bla*_SHV-1_	*armA, mphE, msrE*	3	ybt 9; ICEKp3	1	ST2502	2
2022-KPC-Kpn-37	*bla*_KPC-3_, *bla*_CMY-16,_ *bla*_OXA-10,_ *bla*_SHV-11,_ *bla*_TEM-1D_	*aadA, aadA2, aph (3’)-VIb, aph3-Ia, strA, sul1, sul2, tet(A), floR, catA1, cmlA5, mphA*	3	ybt 9; ICEKp3	1	ST512	1
2022-KPC-Kpn-38	*bla*_KPC-3_, *bla*_SHV-1_	*armA, mphE.v2, msrE*	3	ybt 9; ICEKp3	1	ST2502	2
2022-KPC-Kpn-39	*bla*_KPC-3_, *bla*_SHV-11,_ *bla*_TEM-1D_	*aac(6’)-Ib’, aadA2, aph3-Ia, sul1, catA1, mphA*	2	-	0	ST512	1
2022-KPC-Kpn-40	*bla*_KPC-3_, *bla*_SHV-11,_ *bla*_TEM-1D_	*aadA2, sul1, catA1, mphA*	3	ybt 9; ICEKp3	1	ST512	1
2022-KPC-Kpn-41	*bla*_KPC-3_, *bla*_SHV-1_	*armA, mphE.v2, msrE*	3	ybt 9; ICEKp3	1	ST2502	2
2022-KPC-Kpn-42	*bla*_KPC-3_, *bla*_CMY-16,_ *bla*_OXA-10,_ *bla*_SHV-11,_ *bla*_TEM-1D_	*aadA, aadA2, aph (3’)-VIb, aph3-Ia, strA, sul1, sul2, tet(A), floR, catA1, mphA*	3	ybt 9; ICEKp3	1	ST512	1
2022-KPC-Kpn-43	*bla*_KPC-3_, *bla*_CMY-16,_ *bla*_OXA-10,_ *bla*_SHV-11,_ *bla*_TEM-1D_	*aadA2, aph (3’)-VIb, aph3-Ia, strA, strB, sul1, sul2, tet(A), floR, catA1*	3	ybt 9; ICEKp3	1	ST512	1
2022-KPC-Kpn-44	*bla*_KPC-3_, *bla*_SHV-11,_ *bla*_TEM-1D_	*aadA2, aph3-Ia, sul1, catA1, mphA*	3	ybt 9; ICEKp3	1	ST512	1
2022-KPC-Kpn-45	*bla*_KPC-3_, *bla*_SHV-11,_ *bla*_TEM-1D_	*aadA2, aph3-Ia, sul1, catA1, mphA*	3	ybt 9; ICEKp3	1	ST512	1
2022-KPC-Kpn-46	*bla*_KPC-3_, *bla*_SHV-1_	*armA, mphE.v2, msrE*	3	ybt 9; ICEKp3	1	ST2502	2
2022-KPC-Kpn-47	*bla*_KPC-3_, *bla*_SHV-11,_ *bla*_TEM-1D_	*mphA, aac (6’)-Ib’, aadA2, aph3-Ia, sul1, catA1*	3	-	0	ST512	5
2022-KPC-Kpn-48	*bla*_KPC-3_, *bla*_SHV-11,_ *bla*_TEM-1D_	*sul1, aadA2, catA1, mphA*	3	ybt 9; ICEKp3	1	ST512	1
2022-KPC-Kpn-49	*bla*_KPC-3_, *bla*_SHV-11,_ *bla*_TEM-1D_	*aadA2, aph3-Ia, sul1, catA1, mphA*	3	ybt 9; ICEKp3	1	ST512	1
2022-KPC-Kpn-50	*bla*_KPC-3_, *bla*_SHV-1,_ *bla*_TEM-1D_	*aadA2, aph3-Ia, sul1, catA1, mphA*	3	ybt 9; ICEKp3	1	ST512	1
2022-KPC-Kpn-51	*bla*_KPC-3_, *bla*_SHV-1,_ *bla*_TEM-1D_	*aadA2, sul1, catA1, mphA*	3	ybt 9; ICEKp3	1	ST512	1
2023-KPC-Kpn-52	*bla*_KPC-3,_*bla*_SHV-1_, *bla*_TEM-1D_	*aadA2, aph3-Ia, sul1, catA1, mphA*	0	ybt 9; ICEKp3	1	ST512	1
2023-KPC-Kpn-53	*bla*_KPC-3_, *bla*_CMY-16,_ *bla*_OXA-10_, *bla*_TEM-1D_, *bla*_SHV-1_	*aadA, aadA2, aph(3’)-VIb, aph3-Ia, strA, strB, sul1, sul2, tet(A), floR, catA1, mphA*	3	ybt 9; ICEKp3	1	ST512	1
2023-KPC-Kpn-54	*bla*_KPC-3,_ *bla*_OXA-10,_ *bla*_CTX-M-15_, *bla*_SHV-28,_ *bla*_TEM-1D_	*aac(3)-IIa, aac(6’)-Ib, strA, strB, sul2, CatB4*	2	-	0	ST307	-
2023-KPC-Kpn-55	*bla*_KPC-3_, *bla*_CMY-16,_ *bla*_OXA-10_*, bla*_TEM-1D_, *bla*_SHV-1_	*aadA, aadA2, aph (3’)-VIb, aph3-Ia, strA, strB, sul1, sul2, tet(A), floR, catA1, mphA*	3	ybt 9; ICEKp3	1	ST512	1
2023-KPC-Kpn-56	*bla*_KPC-3_, *bla*_SHV-1_	*aac(6’)-Ib’, aadA2, aph3-Ia, sul1, catA1, mphA*	3	-	0	ST512	5
2023-KPC-Kpn-57	*bla*_KPC-3_, *bla*_SHV-1_	*aadA, ant (2″)-Ia, sul1, catA1*	3	ybt 9; ICEKp3; iuc 1	4	ST101	6
2023-KPC-Kpn-58	*bla*_KPC-3_, *bla*_CMY-16,_ *bla*_OXA-10,_ *bla*_SHV-11,_*bla*_TEM-1D_	*strA, strB, aadA, aadA2, aph (3’)-VIb, aph3Ia, sul1, sul2, tet(A), floR, catA1*	3	ybt 9; ICEKp3	1	ST512	1
2023-KPC-Kpn-59	*bla*_KPC-3_, *bla*_SHV-11,_*bla*_TEM-1D_	*aadA2, sul1, catA1*	3	ybt 9; ICEKp3	1	ST512	1
2023-KPC-Kpn-60	*bla*_KPC-3_, *bla*_SHV-1_*,*	*aadA, ant (2″)-Ia, sul1, catA1*	3	ybt 9; ICEKp3; iuc 1; *rmpA2*	4	ST101	6

^1^ Sequence type (ST) identified by MLST method; ^2^ cluster type (CT) identified by cgMLT method.

## Data Availability

Data can be found in the Excel database created ad hoc, which is archived at the authors’ institution (INMI L. Spallanzani IRCCS, Rome, Italy). The whole-genome sequencing files were submitted to NCBI BioProject ID PRJNA1078116.

## Supplementary Materials

The following supporting information can be downloaded at https://www.mdpi.com/article/10.3390/antibiotics13060561/s1: Table S1: Antimicrobial resistance profiles of *K. pneumoniae* isolates.
