# Supplementary material for: Screening of Klebsiella pneumoniae subsp. pneumoniae Strains with Multi-Drug Resistance and Virulence Profiles Isolated from an Italian Hospital between 2020 and 2023
Source: Antibiotics (Basel). 2024 Jun 15;13(6):561. doi: 10.3390/antibiotics13060561 (PMC11200418; doi:10.3390/antibiotics13060561)
Supplement: Supplementary file 1 [file antibiotics-13-00561-s001.zip › antibiotics-3029204-supplementary.pdf]

**Table S1**. Antimicrobial resistance profiles of *K. pneumoniae* isolates

| | | | | | MIC (mg/L) | | | | | | | | |
|---|---|---|---|---|---|---|---|---|---|---|---|---|---|
| **Strain** | **Sample** | **Year** | ***bla*$_{KPC}$ allele** | **Typing** | **IPM** | **MER** | **MER-VAB** | **CAZ-AVI** | **CEF** | **AMK** | **GEN** | **CIP** | **COL** |
| 2020-KPC-Kpn-1 | BAL | 2020 | KPC-3 | ST512 | **>8 R** | **>8 R** | **0.5 S** | **8 S** | **0.5 S** | **>16 R** | **2 S** | **>1 R** | **2 S** |
| 2020-KPC-Kpn-2 | Rectal swab | 2020 | KPC-3 | ST512 | **>8 R** | **>8 R** | **0.5 S** | **8 S** | **1 S** | **>16 R** | **2 S** | **>1 R** | **2 S** |
| 2020-KPC-Kpn-3 | BC | 2020 | KPC-3 | ST2502 | **8 I** | **8 I** | **0.75 S** | **2 S** | **2 S** | **>16 R** | **≤1 S** | **>1 R** | **0.25 S** |
| 2020-KPC-Kpn-4 | BAL | 2020 | KPC-3 | ST512 | **>8 R** | **>8 R** | **0.5 S** | **4 S** | **2 S** | **8 I** | **2 S** | **>1 R** | **0.25 S** |
| 2020-KPC-Kpn-5 | Rectal swab | 2020 | KPC-3 | ST2502 | **>8 R** | **>8 R** | **0.75 S** | **4 S** | **0.5 S** | **>16 R** | **2 S** | **>1 R** | **0.25 S** |
| 2021-KPC-Kpn-6 | Rectal swab | 2021 | KPC-3 | ST101 | **>8 R** | **>8 R** | **2 S** | **8 S** | **2 S** | **>16 R** | **2 S** | **>1 R** | **0.25 S** |
| 2021-KPC-Kpn-7 | Rectal swab | 2021 | KPC-3 | ST512 | **>8 R** | **>8 R** | **0.19 S** | **4 S** | **2 S** | **16 R** | **4 R** | **>1 R** | **0.25 S** |
| 2021-KPC-Kpn-8 | BC | 2021 | KPC-3 | ST512 | **>8 R** | **>8 R** | **0.38 S** | **4 S** | **2 S** | **16 R** | **2 S** | **>1 R** | **0.25 S** |
| 2021-KPC-Kpn-9 | Rectal swab | 2021 | KPC-3 | ST512 | **>8 R** | **>8 R** | **0.19 S** | **4 S** | **4 R** | **<=4 S** | **>4 R** | **>1 R** | **>16 R** |
| 2021-KPC-Kpn-10 | Rectal swab | 2021 | KPC-3 | ST512 | **>8 R** | **>8 R** | **0.25 S** | **4 S** | **2 S** | **16 R** | **4 R** | **>1 R** | **0.25 S** |
| 2021-KPC-Kpn-11 | Urine | 2021 | KPC-3 | ST512 | **>8 R** | **>8 R** | **0.50 S** | **4 S** | **2 S** | **16 R** | **4 R** | **>1 R** | **0.25 S** |
| 2021-KPC-Kpn-12 | BAL | 2021 | KPC-3 | ST219 | **8 I** | **4 I** | **0.023 S** | **2 S** | **2 S** | **<=4 S** | **≤1 S** | **>1 R** | **0.25 S** |
| 2021-KPC-Kpn-13 | Rectal swab | 2021 | KPC-3 | ST512 | **>8 R** | **>8 R** | **0.19 S** | **8 S** | **1 S** | **16 R** | **4 R** | **>1 R** | **2 S** |
| 2021-KPC-Kpn-14 | Rectal swab | 2021 | KPC-3 | ST745 | **>8 R** | **>8 R** | **0.25 S** | **4 S** | **0.5 S** | **<=4 S** | **≤1 S** | **>1 R** | **0.25 S** |
| 2021-KPC-Kpn-15 | Rectal swab | 2021 | KPC-3 | ST219 | **>8 R** | **8 I** | **0.032 S** | **2 S** | **2 S** | **<=4 S** | **≤1 S** | **>1 R** | **0.25 S** |
| 2021-KPC-Kpn-16 | BAL | 2021 | KPC-3 | ST512 | **>8 R** | **>8 R** | **0.19 S** | **4 S** | **2 S** | **<=4 S** | **>4 R** | **>1 R** | **0.25 S** |
| 2021-KPC-Kpn-17 | BC | 2021 | KPC-3 | ST512 | **>8 R** | **>8 R** | **0.19 S** | **4 S** | **2 S** | **16 R** | **2 S** | **>1 R** | **0.25 S** |
| 2021-KPC-Kpn-18 | Rectal swab | 2021 | KPC-3 | ST512 | **>8 R** | **>8 R** | **1.5 S** | **8 S** | **2 S** | **<=4 S** | **>4 R** | **>1 R** | **0.50 S** |

| | | | | | | | | | | | | | |
|---|---|---|---|---|---|---|---|---|---|---|---|---|---|
| 2021-KPC-Kpn-19 | Rectal swab | 2021 | KPC-3 | ST512 | >8 R | >8 R | 0.125 S | 4 S | 2 S | >16 R | >4 R | >1 R | 0.5 S |
| 2022-KPC-Kpn-20 | BC | 2022 | KPC-3 | ST512 | >8 R | >8 R | 0.75 S | 2 S | 2 S | >16 R | ≤1 S | >1 R | 0.25 S |
| 2022-KPC-Kpn-21 | BC | 2022 | - | ST323 | 2 S | 4 I | 0.023 S | 1/4 S | 0.016 S | ≤4 S | ≤1 S | 0.5 I | 0.25 S |
| 2022-KPC-Kpn-22 | Rectal swab | 2022 | KPC-3 | ST512 | >8 R | >8 R | 0.032 S | 1/4 S | 2 S | >16 R | 4 R | >1 R | 0.25 S |
| 2022-KPC-Kpn-23 | BC | 2022 | KPC-3 | ST512 | >8 R | >8 R | 0.5 S | 8/4 S | 1.5 S | ≤4 S | ≤1 S | >1 R | 0.25 S |
| 2022-KPC-Kpn-24 | BAL | 2022 | KPC-3 | ST512 | >8 R | >8 R | 1.5 S | 8/4 S | 2 S | ≤4 S | ≤1 S | >1 R | 0.25 S |
| 2022-KPC-Kpn-25 | Rectal swab | 2022 | KPC-3 | ST512 | >8 R | >8 R | 0.016 S | 2/4 S | 1.5 S | >16 R | >4 R | >1 R | 0.25 S |
| 2022-KPC-Kpn-26 | Urine | 2022 | KPC-3 | ST512 | >8 R | >8 R | 1.0 S | 3 S | 2 S | 2 S | >4 R | >1 R | 0.25 S |
| 2022-KPC-Kpn-27 | BAL | 2022 | KPC-3 | ST512 | >8 R | >8 R | 0.75 S | 8/4 S | 2 S | ≤4 S | ≤1 S | >1 R | 0.25 S |
| 2022-KPC-Kpn-28 | BAL | 2022 | KPC-3 | ST1519 | >8 R | >8 R | 0.38 S | 8/4 S | 2 S | 16 R | >4 R | >1 R | 0.25 S |
| 2022-KPC-Kpn-29 | Rectal swab | 2022 | KPC-19 | ST512 | >8 R | >8 R | 8 S | 12 R | 8 R | ≤4 S | ≤1 S | >1 R | 0.25 S |
| 2022-KPC-Kpn-30 | BC | 2022 | KPC-3 | ST512 | >8 R | >8 R | 1.5 S | 8/4 S | 2 S | >16 R | ≤1 S | >1 R | 0.25 S |
| 2022-KPC-Kpn-31 | Urine | 2022 | KPC-3 | ST512 | >8 R | >8 R | 0.75 S | 6 S | 2 S | >16 R | ≤1 S | >1 R | 0.25 S |
| 2022-KPC-Kpn-32 | BAL | 2022 | KPC-3 | ST512 | >8 R | >8 R | 4 S | 6 S | 2 S | >16 R | ≤1 S | >1 R | 0.25 S |
| 2022-KPC-Kpn-33 | Rectal swab | 2022 | KPC-3 | ST512 | >8 R | >8 R | 6 S | 4 S | 1 S | >16 R | ≤1 S | >1 R | 0.25 S |
| 2022-KPC-Kpn-34 | Rectal swab | 2022 | KPC-3 | ST512 | >8 R | >8 R | 2 S | 8/4 S | 1.5 S | ≤4 S | ≤1 S | >1 R | 0.50 S |
| 2022-KPC-Kpn-35 | Urine | 2022 | KPC-3 | ST512 | >8 R | >8 R | 3 S | 6 S | 2 S | 32 R | ≤1 S | >1 R | 0.25 S |
| 2022-KPC-Kpn-36 | BC | 2022 | KPC-3 | ST2502 | >8 R | >8 R | 0.5 S | 4/4 S | 2 S | >16 R | >4 R | >1 R | 0.50 S |
| 2022-KPC-Kpn-37 | Rectal swab | 2022 | KPC-3 | ST512 | >8 R | >8 R | 8 S | 2 S | 2 S | >16 R | ≤1 S | >1 R | 0.25 S |
| 2022-KPC-Kpn-38 | Rectal swab | 2022 | KPC-3 | ST2502 | >8 R | >8 R | 1.5 S | 3 S | 2 S | >16 R | >4 R | >1 R | 0.25 S |
| 2022-KPC-Kpn-39 | Urine | 2022 | KPC-3 | ST512 | 8 R | <2 S | 0.032 | 2/4 S | 4 R | >16 R | 2 S | >1 R | 0.25 S |
| 2022-KPC-Kpn-40 | BAL | 2022 | KPC-3 | ST512 | 8 R | 8 R | 0.5 S | 8/4 S | 4 R | ≤4 S | ≤1 S | >1 R | 0.25 S |
| 2022-KPC-Kpn-41 | Rectal swab | 2022 | KPC-3 | ST2502 | 8 R | 8 R | 0.5 S | 3 S | 2 S | >16 R | >4 R | >1 R | 0.25 S |
| 2022-KPC-Kpn-42 | BAL | 2022 | KPC-3 | ST512 | >8 R | >8 R | 6 S | 4 S | 2 S | >16 R | ≤1 S | >1 R | 0.25 S |

| | | | | | | | | | | | | | |
|---|---|---|---|---|---|---|---|---|---|---|---|---|---|
| 2022-KPC-Kpn-43 | Urine | 2022 | KPC-3 | ST512 | **>8 R** | **>8 R** | **8 S** | **6 S** | **1.5 S** | **>16 R** | **≤1 S** | **>1 R** | **0.25 S** |
| 2022-KPC-Kpn-44 | BC | 2022 | KPC-3 | ST512 | **>8 R** | **>16 R** | **16 R** | **1 S** | **0.5 S** | **≤4 S** | **≤1 S** | **>1 R** | **0.25 S** |
| 2022-KPC-Kpn-45 | BAL | 2022 | KPC-3 | ST512 | **>8 R** | **>16 R** | **4/8 S** | **8/4 S** | **1 S** | **≤4 S** | **≤1 S** | **>1 R** | **0.50 S** |
| 2022-KPC-Kpn-46 | Rectal swab | 2022 | KPC-3 | ST2502 | **>8 R** | **>16 R** | **4/8 S** | **8/4 S** | **2 S** | **>16 R** | **>4 R** | **>1 R** | **0.25 S** |
| 2022-KPC-Kpn-47 | BAL | 2022 | KPC-3 | ST512 | **>8 R** | **>16 R** | **≤2/8 S** | **1/4 S** | **0.25 S** | **>16 R** | **2 S** | **>1 R** | **0.25 S** |
| 2022-KPC-Kpn-48 | BC | 2022 | KPC-3 | ST512 | **>8 R** | **>16 R** | **≤2/8 S** | **4/4 S** | **4 R** | **≤4 S** | **≤1 S** | **>1 R** | **0.25 S** |
| 2022-KPC-Kpn-49 | Rectal swab | 2022 | KPC-3 | ST512 | **>8 R** | **>16 R** | **≤2/8 S** | **8/4 S** | **1 S** | **≤4 S** | **≤1 S** | **>1 R** | **0.25 S** |
| 2022-KPC-Kpn-50 | Rectal swab | 2022 | KPC-3 | ST512 | **>8 R** | **>16 R** | **≤8/8 S** | **8/4 S** | **4 R** | **≤4 S** | **≤1 S** | **>1 R** | **0.25 S** |
| 2022-KPC-Kpn-51 | Rectal swab | 2022 | KPC-3 | ST512 | **>8 R** | **>16 R** | **≤2/8 S** | **2/4 S** | **2 S** | **≤4 S** | **≤1 S** | **>1 R** | **0.25 S** |
| 2023-KPC-Kpn-52 | Rectal swab | 2023 | KPC-3 | ST512 | **>8 R** | **>16 R** | **8/8 S** | **8 S** | **2 S** | **≤4 S** | **≤1 S** | **>1 R** | **0.25 S** |
| 2023-KPC-Kpn-53 | Urine | 2023 | KPC-3 | ST512 | **>8 R** | **>16 R** | **> 8/8 S** | **6 S** | **2 S** | **>16 R** | **≤1 S** | **>1 R** | **0.25 S** |
| 2023-KPC-Kpn-54 | Rectal swab | 2023 | KPC-3 | ST307 | **>8 R** | **>16 R** | **≤2/8 S** | **4/4 S** | **1 S** | **≤4 S** | **>4 R** | **>1 R** | **0.25 S** |
| 2023-KPC-Kpn-55 | Rectal swab | 2023 | KPC-3 | ST512 | **>8 R** | **>16 R** | **> 8/8 S** | **8 S** | **2 S** | **>16 R** | **≤1 S** | **>1 R** | **0.25 S** |
| 2023-KPC-Kpn-56 | Rectal swab | 2023 | KPC-3 | ST512 | **>8 R** | **>16 R** | **≤2/8 S** | **<=0.25/4 S** | **1 S** | **>16 R** | **2 S** | **>1 R** | **0.25 S** |
| 2023-KPC-Kpn-57 | Rectal swab | 2023 | KPC-3 | ST101 | **>8 R** | **>16 R** | **8/8 S** | **4/4 S** | **1 S** | **≤4 S** | **>4 R** | **>1 R** | **16 R** |
| 2023-KPC-Kpn-58 | BC | 2023 | KPC-3 | ST512 | **>8 R** | **>16 R** | **> 8/8 S** | **8 S** | **2 S** | **>16 R** | **≤1 S** | **≤0.125 S** | **0.25 S** |
| 2023-KPC-Kpn-59 | Rectal swab | 2023 | KPC-3 | ST512 | **>8 R** | **>16 R** | **≤2/8 S** | **4/4 S** | **2 S** | **≤4 S** | **≤1 S** | **>1 R** | **0.25 S** |
| 2023-KPC-Kpn-60 | BC | 2023 | KPC-3 | ST101 | **>8 R** | **>16 R** | **≤2/8 S** | **8/4 S** | **0.5 S** | **≤4 S** | **>4 R** | **>1 R** | **0.25 S** |

**Table S1**
S, Susceptible; R, resistant, I sensible increased exposure;
**AMK**, Amikacina; **CIP**, Ciprofloxacina; **GEN**, Gentamicina; **IPM**, Imipenem; **MER**, Meropenem; **COL**: Colistina, **CZA**, Ceftazidime-avibactam; **MER-V**, Meropenem-vaborbactam; **CEF**, Cefiderocol; **S**, susceptible; **R**, resistant; **BAL**, bronchoalverolar lavage; **BC** Blood culture
